# Supplementary material for: Mapping protein interactions of sodium channel NaV1.7 using epitope‐tagged gene‐targeted mice
Source: EMBO J. 2018 Jan 15;37(3):427–45. doi: 10.15252/embj.201796692 (PMC5793798; doi:10.15252/embj.201796692)
Supplement: Supplementary file 1 — Expanded View Figures PDF [file EMBJ-37-427-s001.pdf]

Expanded View Figures

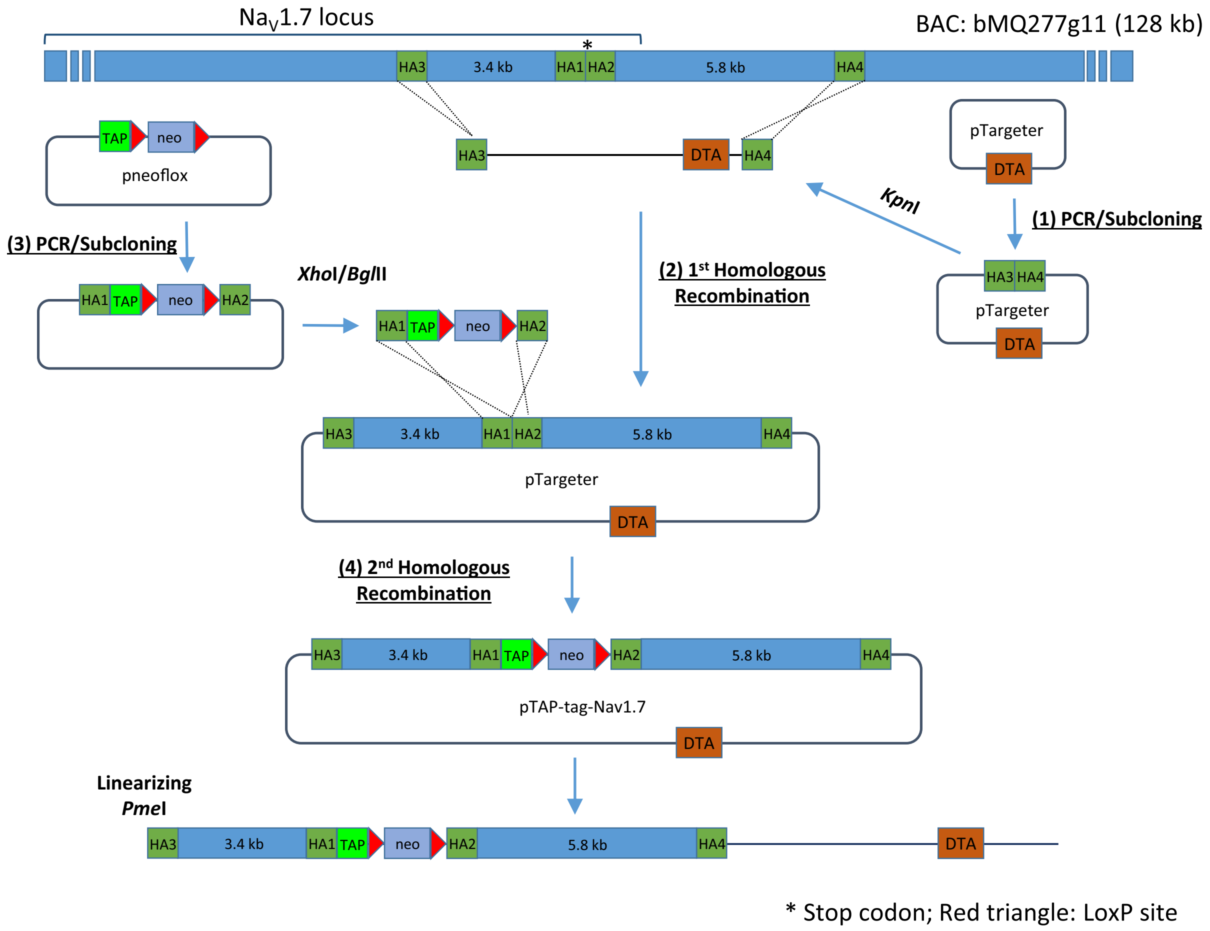

Figure EV1. Schematic diagrams showing the steps for constructing a TAP-tagged Na$_V$1.7 gene targeting vector using BAC homologous recombineering method.

Step (1), two short homology arms HA3 and HA4 were amplified by PCR and then inserted into a retrieval vector pTargeter. Step (2), a 9.1-kb genomic DNA fragment (3.4 kb plus 5.8 kb) was retrieved from BAC clone bMQ277g11 through homologous recombineering. Step (3), homology arms HA1 and HA2 were amplified and subcloned into pneoflox vector. Step (4), the excised TAP-tag cassette was inserted into the pTargeter-HA3-HA4 vector by homologous recombineering in EL250 cells. The targeting vector was linearised with *Pme*I restriction enzyme and was used to generate TAP-tagged Na$_V$1.7 mouse.

 © 2018 The Authors

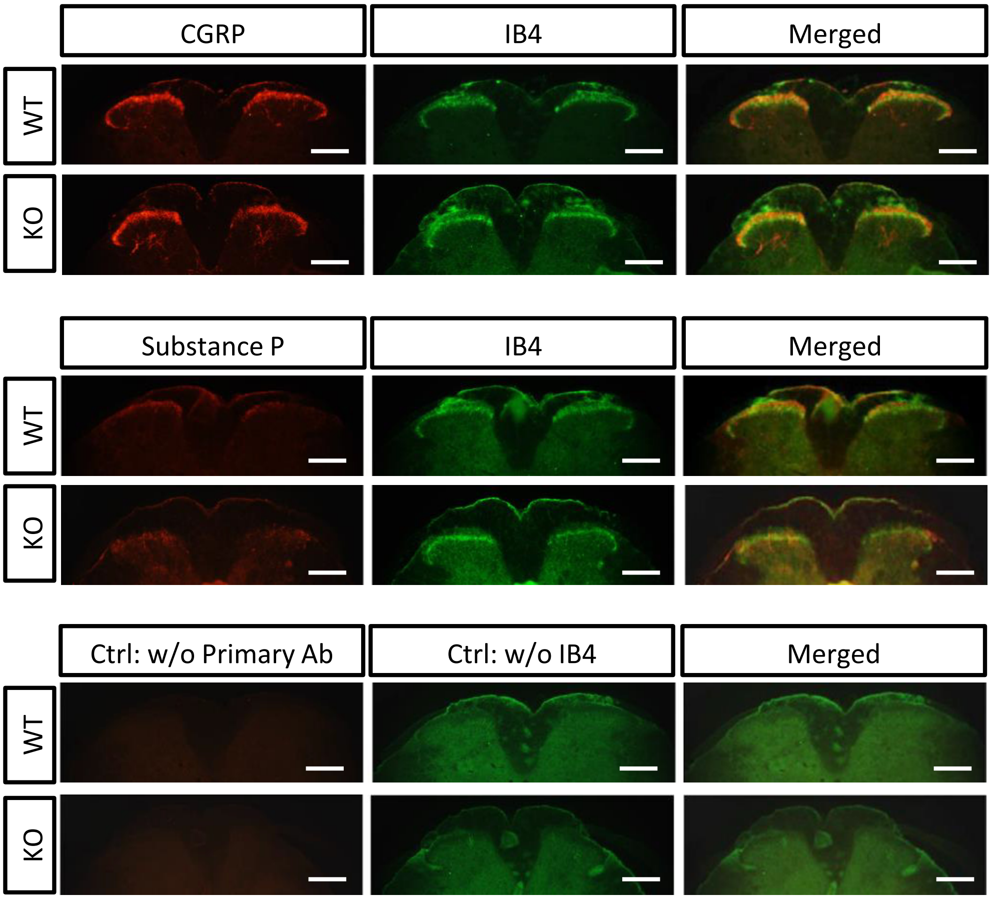

Figure EV2. Immunohistochemistry of spinal cords.

Cross sections of lumbar spinal cord of Na$_V$1.7 knockout mice (KO) and littermate wild-type control mice (WT) were labelled with anti-CGRP (in red), anti-substance P (in red) and IB4 (in green). Right panel: left panel merged to middle panel. Scale bar = 250 μm.

© 2018 The Authors
