# Supplementary figures and images for: Mapping protein interactions of sodium channel NaV1.7 using epitope‐tagged gene‐targeted mice
Source: EMBO J. 2018 Jan 15;37(3):427–45. doi: 10.15252/embj.201796692 (PMC5793798; doi:10.15252/embj.201796692)

Figure 5 – Source Data

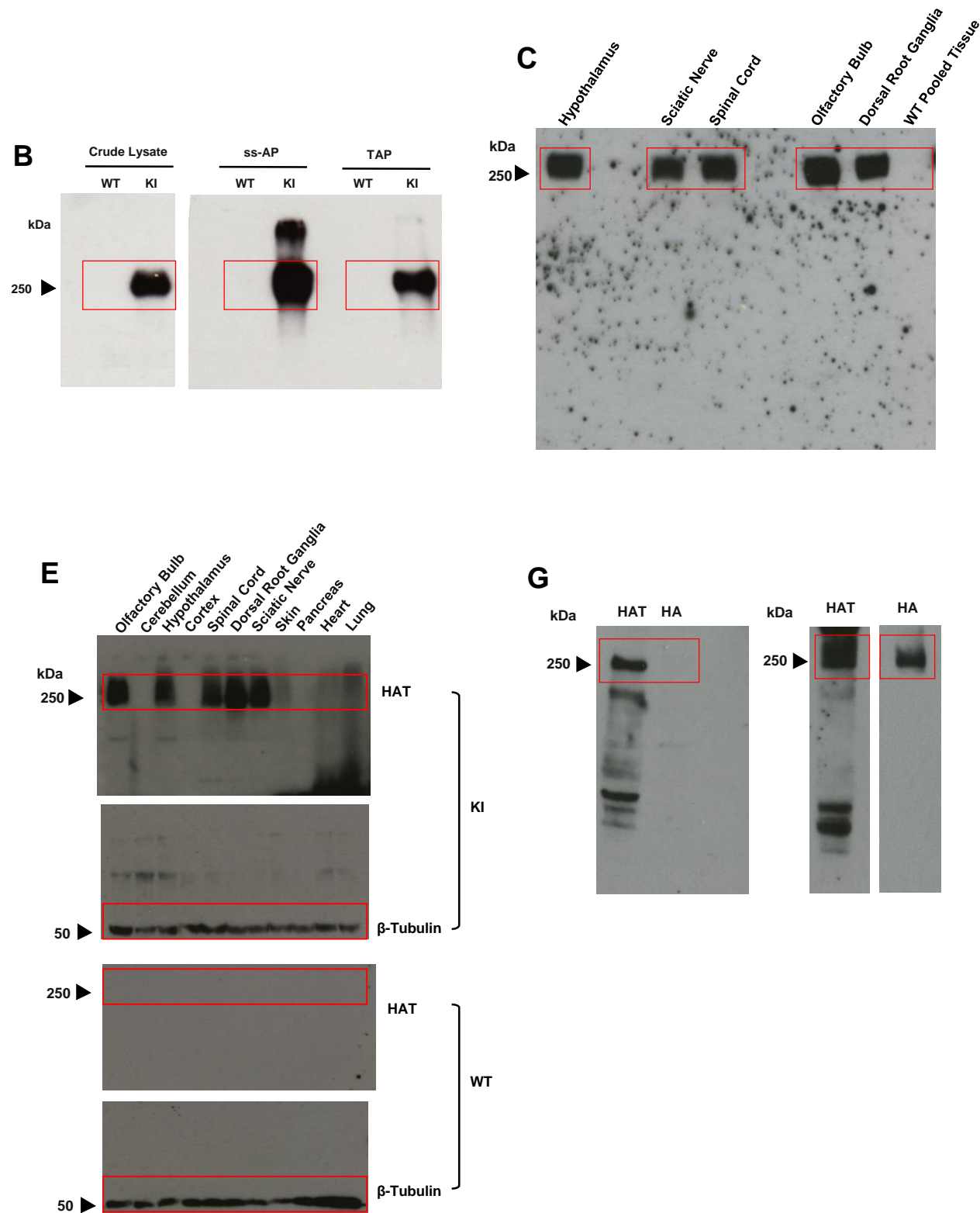

Supplement: Supplementary file 4 — Source Data for Figure 5 [file EMBJ-37-427-s003.pdf]
